# Small intestinal neuroendocrine tumors lack early genomic drivers, acquire DNA repair defects and harbor hallmarks of low *REST* expression

**DOI:** 10.1038/s41598-025-01912-4

**Published:** 2025-05-23

**Authors:** Felix Bolduan, Niklas Müller-Bötticher, Olivia Debnath, Ines Eichhorn, Yvonne Giesecke, Alexandra Wetzel, Shashwat Sahay, Tomasz Zemojtel, Marten Jaeger, Ute Ungethuem, Christoph Roderburg, Catarina Alisa Kunze, Annika Lehmann, David Horst, Frank Tacke, Roland Eils, Bertram Wiedenmann, Michael Sigal, Naveed Ishaque

**Affiliations:** 1 Department of Hepatology & Gastroenterology, Charité Universitätsmedizin Berlin, Campus Virchow-Klinikum and Campus Charité Mitte, 13353 Berlin, Germany; 2 BIH Charité Junior Digital Clinician Scientist Program, Berlin Institute of Health at Charité – Universitätsmedizin Berlin, BIH Biomedical Innovation Academy, Charitéplatz 1, 10117 Berlin, Germany; 3 Center of Digital Health, Berlin Institute of Health at Charité Universitätsmedizin Berlin, Charitéplatz 1, 10117 Berlin, Germany; 4 Department of Mathematics and Computer Science, Freie Universität Berlin, Arnimallee 14, 14195 Berlin, Germany; 5 Core Facility Genomics, Berlin Institute of Health at Charité - Universitätsmedizin Berlin, Charitéplatz 1, 10117 Berlin, Germany; 6 Department of Gastroenterology, Hepatology and Infectious Diseases, University Hospital Düsseldorf, Medical Faculty of Heinrich Heine University Düsseldorf, Düsseldorf, Germany; 7 Institute of Pathology, Charité - Universitätsmedizin Berlin, Corporate Member of Freie Universität Berlin and Humboldt Universität zu Berlin, 10117 Berlin, Germany; 8 German Cancer Consortium (DKTK), Partner Site Berlin, CCCC (Campus Mitte), Berlin, Germany; 9 Berlin Institute for Medical Systems Biology, Hannoversche Straße 28, 10115 Berlin, Germany

**Keywords:** Computational biology and bioinformatics, Cancer genomics, Cancer, Gastrointestinal cancer

## Abstract

The tumorigenesis of small intestinal neuroendocrine tumors (siNETs) is not understood and comprehensive genomic and transcriptomic data sets are limited. Therefore, we performed whole genome and transcriptome analysis of 39 well differentiated siNET samples. Our genomic data revealed a lack of recurrent driver mutations and demonstrated that multifocal siNETs from individual patients can arise genetically independently. We detected germline mutations in Fanconi anemia DNA repair pathway (FANC) genes, involved in homologous recombination (HR) DNA repair, in 9% of patients and found mutational signatures of defective HR DNA repair in late-stage tumor evolution. Furthermore, transcriptomic analysis revealed low expression of the transcriptional repressor REST. Summarizing, we identify a novel common transcriptomic signature of siNETs and demonstrate that genomic alterations alone do not explain initial tumor formation, while impaired DNA repair likely contributes to tumor evolution and represents a potential pharmaceutical target in a subset of patients.

## Introduction

Small intestinal neuroendocrine tumors (siNETs) are the most frequent form of small bowel cancer, overall slow-growing, well-differentiated and often diagnosed in a metastatic state, precluding curative treatment options. In such a palliative setting, systemic treatment is needed, however, the number of available therapeutic options is limited^1^. For the development of novel precision treatment options, robust knowledge of the tumor biology is mandatory. As cancer is in general a disease of the genome^2^, its alterations are key features to understand the tumor biology. Previous studies have investigated and described the genomic alterations of siNETs^3–11^.

First, siNETs exhibit a broad range of chromosomal amplifications and deletions including the loss of chromosome 18 and amplifications of chromosomes 4, 5, 14, and 20, but so far, their tumorigenic function remains unidentified^12,13^. In a single study, amplifications in genes of the PI3K/AKT/mTOR signaling pathway were described in 14 out of 48 siNETs (29%) explaining the partial response of mTOR inhibitors in siNET treatment^3^.

Furthermore, siNETs are in general mutationally quiet and microsatellite stable^6,7,10,14^. *CDKN1B* mutations have been considered as potential drivers in siNET development^4–8,10^. *CDKN1B* encodes for p27 and germline mutations are associated with the multiple endocrine neoplasia (MEN) syndrome MEN4^15^. Nonetheless, given the relatively low frequency of *CDKN1B* alterations (8%^4^, 8.5%^8^, 11%^5^, 13%^6^ and 23%^7^ of siNET samples), its general role as a recurrent driver of siNET tumorigenesis is not clear. Additionally, alterations in the *TNRC6B* and *CDKN2A* genes as possible tumor suppressors were observed in a small subset of siNETs^5,7,10^. Recently, a whole genome sequencing study of siNETs revealed driver mutations like *TP53*, *RB1*, *KRAS*, *NRAS*, and *MET* mutations^10^. However, this study included also G3 tumors which display a different biology and therefore genetic profile than G1/G2 siNETs.

The transcriptome of siNETs has not been extensively studied, and only a limited amount of data exists^16–21^. These studies elucidated different expression profiles within siNETs^16,17^ or between primary tumor and metastases^19,20^. With one exception, a combined analysis of genomic and transcriptomic alterations has not been performed yet. Only Postel et al*.* studied the genome and transcriptome of five siNET paired samples consisting of liver metastases and primary tumors^19^. They identified several differentially expressed genes and pathways between primary tumor and metastasis but without modelling the differential sampling sites or tumor cell content of samples, and so far without further implications.

Here, we provide the largest study of a combined genome and transcriptome analyses of siNETs. We performed whole genome and bulk RNA sequencing of 39 siNET samples derived from 32 patients of our NET clinical center. Consistent with previous results, we detected chromosomal aberrations like deletion of chromosome 18. We observed no strong common driver mutations and by analyzing paired tumor samples found that multifocal tumors are genetically independent^5,6^ thus we conclude that no somatic genomic event causes initial siNET formation. While we observed mutational signatures of defective DNA repair in the majority of samples, these were traced to later in evolution, therefore not sufficient to be considered as early driver events. Furthermore, we identified germline predisposition mutations of Fanconi anemia genes, which are involved in DNA repair, in 9% of our patients. These data together suggest that tumor formation of siNETs is not driven by somatic genomic events alone, but impaired DNA repair likely contributes to diseases progression and provides arguments for targeting defective homologous recombination in a subset of patients. To characterize the result of a yet unknown tumor initiating event, we performed RNA sequencing and revealed a novel transcriptomic signature of siNETs defined by low expression of the transcription repressor REST. Our data provide novel molecular insights into pathogenesis of siNETs and may be useful for identifying patients at risk for advanced siNET formation.

## Results

### Mutational signatures implicate low mutational burden in siNETs

First, we established a cohort of clinically well characterized samples from 32 siNET patients from which neoplastic and adjacent healthy tissue was obtained (Supplementary Table 1). In total we profiled 39 samples, of which there were 22 siNET primary tumors (median Ki67 2%), 12 hepatic metastases of siNETs (median Ki67 5%), 4 lymph node metastases and 1 peritoneal metastasis. All samples were reviewed by pathologists and classified as neuroendocrine tumors G1/G2.

**Fig. 1:**
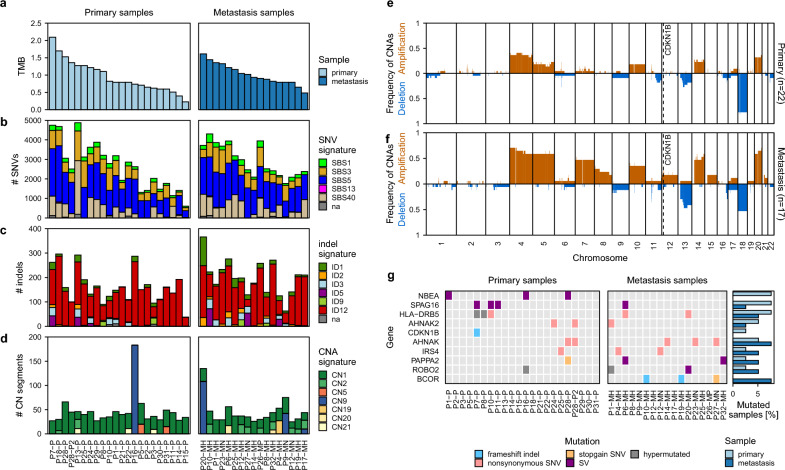
Mutational landscape of siNETs. (**a**) Tumor mutational burden (TMB) of each sample as somatic SNVs and InDels in coding regions divided over the summed lengths of distinct non-overlapping coding regions. (**b**–**d**) COSMIC mutational signatures for (**b**) single nucleotide variations (SNVs), (**c**) indels, and (**d**) copy number aberrations (CNAs). (**e**, **f**) CNA landscape in siNETs of (**e**) primary tumors and (**f**) metastasis. CDKN1B location is highlighted. (**g**) Recurrently mutated genes (3 or more patients) in siNETs and CDKN1B mutations. *P* primary tumor, *MH* hepatic metastasis, *MN* lymph node metastasis, *MP* peritoneal metastasis.

DNA and RNA were isolated from the tumor and normal samples and subjected to library preparation and short read sequencing-by-synthesis. DNA sequencing yielded high quality data (Fig. S1a, Supplementary Table 2) with an average genome coverage of 85 × in tumors and 51 × in matched controls and an average of 308 million RNA-seq read pairs (Supplementary Table 3), followed by identification of somatic mutations. Comparable to other studies of siNETs we observed a low number of somatic mutations^6,7^, which also corresponded to a low tumor mutational burden (mean ± sem: 0.98 ± 0.06, median: 0.88 mutations per Mb coding sequence, Fig. 1a).

When analyzing the COSMIC single base substitution (SBS) mutational signatures^22^ we mainly detected SBS5 (unknown aetiology/clock-like), SBS3 (defective homologous recombination DNA repair), SBS40 (unknown aetiology/age-related), SBS1 (spontaneous deamination 5-methylcytosine to thymine/clock-like) and to a very little extent SBS13 (APOBEC activity) (Fig. 1b). This is in line with previous SBS mutational signature analyses of siNETs^7^ and shows the relatively mutationally quiet nature of siNETs with aging related signatures being the most prominent. The presence of SBS signature 3 may indicate a role for defective HR DNA repair in siNET tumorigenesis or progression.

Similar to the low SNV burden in our samples, we also observed a low number of indels (mean ± sem: 183 ± 11, median: 176, Fig. 1c). We investigated COSMIC indel (ID) mutational signatures^22^ in our samples. The most prominent signature was ID12, which is defined by deletions of 2 base pairs (bp) in ≥ 6 bp number repeat units. The aetiology of ID12 is unknown, and it was observed to a little extent in prostate adenocarcinoma but not in endocrine pancreatic tumors^22^. Signatures ID1 (aetiology: slippage during DNA replication of the replicated DNA strand), ID2 (aetiology: slippage during DNA replication of the template DNA strand), ID3 (tobacco smoking), ID5 (unknown aetiology) and ID9 (unknown aetiology) were also found in our cohort but with a very low extent when compared to other tumor entities^22^.

Recently, signatures of copy number alteration (CNAs) in human cancer were published^23^. Although these signatures were originally derived from SNP array data, we analyzed our WGS data for these CNA signatures (Fig. 1d). Again, we detected a relatively low mutational exposure, with the diploid signature 1 being most prominent. In metastases the portion of the tetraploid CNA signature 2 was higher. In four samples we detected signature 9, a signature of chromosomal instability. Further analyses could demonstrate that in two of these tumors chromothripsis occurred (P16 and P20). Chromothripsis as a chromosomal catastrophe with chromosomal shattering and rearrangement was previously described in siNETs on chromosome 12 and 13^6,7^. Interestingly, it also occurred in one of our samples on chromosome 13 (P16). The other sample (P20) displayed chromothripsis on the p-arm of chromosome 9. Additionally, we detected signature 21 with unknown aetiology. This signature 21 was found in 5 samples and was previously exclusively described in pheochromocytoma and paraganglioma, both neuroendocrine tumor entities. Although the proportion of this signature is quite low, it only seems to appear in neuroendocrine tumor entities and may be specific for them. Further understanding of the aetiology of CNA signature 21 could provide new insights in the tumorigenesis and/or tumor propagation of these neoplasms.

### Chromosomal aberrations of siNETs are enriched in metastasis

We further investigated the CNAs in our siNET cohort (Fig. 1e,f). We detected gains on chromosome 4, 5, 7, 10, 14, 17 and 20 and losses on chromosome 6, 9, 11, 13, 16 and 18, which is in line with previous reports^3,4,7,24^. Chromosomal aberration, especially gains, are more frequent in metastases, which is consistent with a higher amount of the tetraploid CNA signature 2 in metastases described earlier, indicating that chromosomal aberrations accumulate over time. Interestingly, only chromosome 18 deletions were observed less frequently in metastatic (50%) tumors compared to primaries (68%), suggesting that chromosome 18 deletion is not necessary for the evolution of metastases but gives an advantage on primary site. In accordance with this postulation, one of our patients (P1) displayed loss of chromosome 18 in the primary tumor but not in the metastasis.

We also checked for differences in telomere lengths between primary tumors and metastases in our cohort, but we could not detect telomere lengthening in our samples nor any significant alteration between primary tumor and metastases (Fig. S2).

### Most siNETs lack strong candidates for early genomic drivers

Prior studies stated that *CDKN1B* gene mutations are the most common driver mutation in siNETs with a frequency between 8%^4,8^ and 23%^7^ of samples. We only detected a single *CDKN1B* non-silent mutation in our samples. Recently, TNRC6B mutations have been described as recurrent mutations, detected in 8% of siNET samples^5^. We did not observe *TNRC6B* gene mutations in our cohort.

As possible oncogenic mutations, we observed *AHNAK* and *BCOR* gene mutations with a higher frequency in metastasis samples (Fig. 1g). Both genes encode for known tumor suppressors, the first involved in the TGFβ signaling pathway via R-Smad mediated downregulation of cell cycle^25^, the latter in suppression of cell cycle and proliferation via Myc inhibition^26,27^. *AHNAK* mutations have been reported in other entities of neuroendocrine neoplasms (neuroblastomas and adrenocortical carcinomas, both with a frequency below 5%^28^), whereas *BCOR* mutations have been previously found in siNETs with a frequency of 5.6%^29^. However, we observed *AHNAK* and *BCOR* mutations only with a low recurrence and to a smaller extent in primary tumors. This suggests that they are acquired during tumor progression rather than drivers of the initial oncogenic transformation.

We identified mutations on non-coding RNA (ncRNA) (Fig. S3). Although it is difficult to interpret their functional effect on the gene product, their rarity and inconsistency among multiple tumors from the same patient (P10 and P28) suggest that ncRNA mutations are unlikely drivers of siNET emergence.

In conclusion, we did not observe any frequent gene mutation that explains the initial oncogenic transformation in most siNET samples.

### siNET evolution implicates late-stage defective homologous repair

**Fig. 2:**
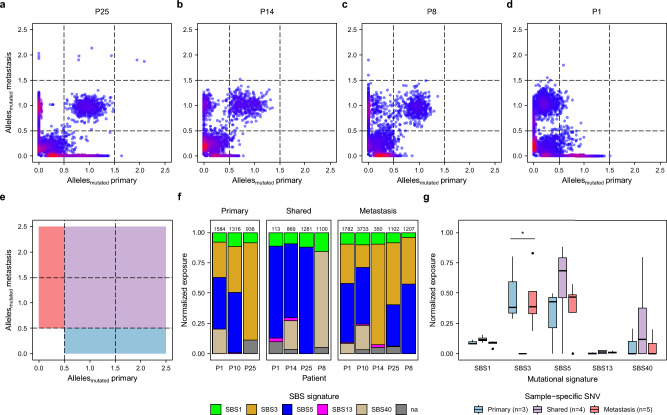
Evolution of siNETs and their mutational processes. (**a**–**d**) Scatter plots of the predicted number of mutated alleles that are shared and sample-specific for matched primary and metastasis. (**e**) Definition of thresholds for predicted number of mutated alleles to determine shared (purple), primary-specific (blue), and metastasis-specific (red) mutations of the major clone. (**f**, **g**) Mutational signatures of shared and primary/metastasis-specific mutations. (**f**) Number of SNVs is indicated above each bar. (**g**) Tested using Kruskall-Wallis, *: *p* < 0.05.

Our cohort included seven pairs of samples each from one patient allowing us to investigate the siNET heterogeneity and evolution by analyzing shared mutations of these tumors (Fig. 2a–d). Four pairs (P25 primary tumor and metastases (Fig. 2a), P14 primary tumor and metastases (Fig. 2b), P8 primary tumor and lymph node metastasis (Fig. 2c), P12 lymph node and hepatic metastasis (Fig. S4g) showed a substantial number of shared mutations indicating a clear relationship for each pair originating from a common ancestral clone (Fig. S4h). Mutations exclusively occurring either in the metastasis or the primary tumor demonstrate an ongoing divergent evolution.

In one pair (P1 primary tumor and hepatic metastasis (Figs. 2d and S4d,h)) only ~ 1.5% of the metastatic clonal mutations were shared with the primary tumor. Modelling the mutated allele counts revealed that the metastatic lesion was derived from a primary subclone (~ 21% clonality). Consistent with this finding of subclonal heterogeneity, we detected chromosome 18 loss only in the primary tumor but not in metastases, implying that the minor subclone giving rise to the metastasis did not harbor the chromosome 18 deletion.

As previously mentioned, we detected SBS3, a mutational signature of defective HR DNA repair in our samples (Figs. 2e–g, S4i). To investigate if these mutations arise early in tumorigenesis, we took advantage of our genetically related matched pairs and analyzed SBS signatures from both the shared mutations and the primary/metastasis-specific mutations. Mutations that occur exclusively in either primary or metastatic lesions are acquired after formation of the most recent common ancestor of the tumor, whereas shared mutations occur early, before divergent evolution. Interestingly, SBS3 was not detected in the shared mutations but was present in the mutations specific to primaries or metastasis, suggesting that this signature is a late acquired characteristic of siNET evolution, consistent with observations in colorectal cancer^30^. However, the precise role of the SBS signature in siNETs remains unclear, given the low mutational burden and lack of other genome instability indicators.

In two pairs of samples (P28 two primary tumors and P10 primary tumor and hepatic metastasis) we found only a very limited number of shared mutations indicating that these tumors developed independently (Fig. S4). This is in line with previous results and emphasizes that in certain, yet unknown conditions siNETs can develop independently out of different progenitors^5,6,31^.

To assess if this condition is associated with bacterial infection, we screened for bacterial DNA but did not detect any specific amount of foreign DNA in our tumor samples, though our sequencing protocols are not necessarily suitable for bacterial DNA isolation. Previous studies have shown that colibactin, a genotoxin found in certain *E. coli* strains, has a significant impact on the development of colorectal cancer and leaves a specific mutational signature^32^. However, we did not find residual mutational signatures of colibactin induced DNA mutations in our samples, arguing against an involvement of colibactin in siNET formation.

Previously, it has been stated that loss of imprinting (LOI) of *IGF2* is a condition that enables siNET development^31^ however, allele-specific expression analysis in our samples only supports *IGF2* LOI in few samples (Fig. S5).

### Multi-focal tumors associated with pathogenic germline mutations in DNA repair genes﻿

Next, we checked if germline mutations confer predisposition that enables siNET formation in a broader region of intestine. Interestingly, among our 32 siNET patients, three possessed ClinVar annotated pathogenic or likely pathogenic germline mutations in genes that belong to the Fanconi anemia DNA repair pathway (FANC genes). These mutations include a likely pathogenic *FANCA* mutation (rs769862233) in patient P19, a pathogenic *FANCC* mutation (rs104886459) in patient P12, and a pathogenic *FANCM* mutation (rs757391108) in patient P25 (Supplementary Table 4). The *FANCA* mutation was a point mutation in the splice acceptor site, and the *FANCC* and *FANCM* mutations were deletions that resulted in frameshifts. The mutations are present at extremely low frequencies in the population (allele freqnecies of 0.00004 and 0.00001 in ExAC for the *FANCA* and *FANCM* mutations, respectively). We did not observe any further pathogenic or likely pathogenic germline predispositions in our cohort. Interestingly, all three patients had multifocal siNETs. *FANCA*, *FANCC*, and *FANCD* genes encode for proteins that are part of the Fanconi anemia core complex, responsible for detection of DNA interstrand cross-links^33^ and initiation of their repair with the help of the HR machinery^34,35^.

Germline mutations in siNETs have been described previously with a frequency of 9–11% and include mutations in *ATM*, *RAD51C*, *MUTYH*, and *BLM*^36,37^. Interestingly, all four genes encode for known tumor suppressors involved in DNA repair^33,38–40^. Taken together, defective HR DNA repair seems to be a feature of siNET biology and could explain the broad range of chromosomal aberrations although the precise mechanism remains uncertain.

In summary, our comprehensive genome analyses did not reveal commonly mutated genes. Furthermore, multifocal siNETs arose independently. Together, these observations indicate that initial siNET formation is not driven by a genetic event but rather by an unknown condition that affects a broader region of the intestine. However, impaired DNA repair seems to be a recurrent signature in siNET evolution as demonstrated by germline mutations in FANC genes and the occurrence of the defective HR DNA repair signature SBS3 in late acquired mutations.

### Low REST expression is a hallmark of siNETs

**Fig. 3:**
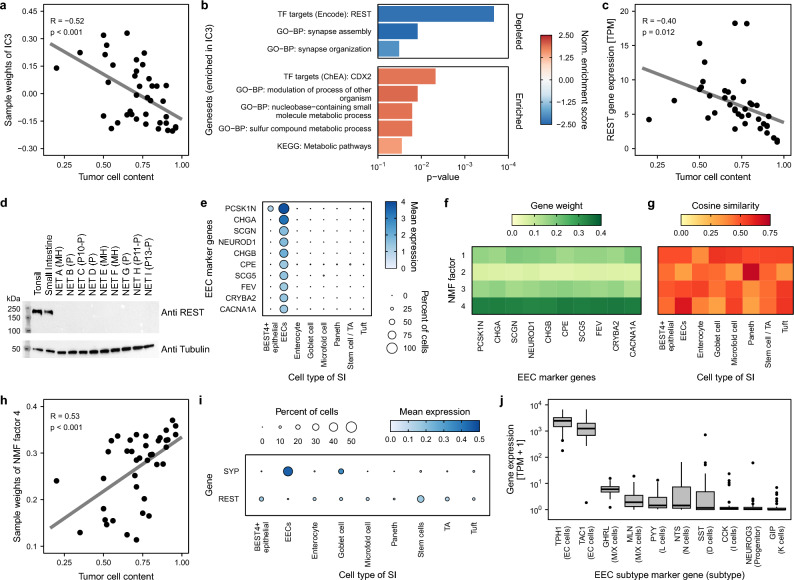
siNET gene expression resembles enteroendocrine cells. For deconvolution of tumor and non-tumor gene expression signatures independent component analysis was performed. (**a**) Independent component 3 of siNET expression anti-correlates with tumor cell content and (**b**) is depleted in REST target genes (*GO-BP* gene ontology biological process, *TF* transcription factor, *p*-values are adjusted using the Benjamini–Hochberg procedure). The top enriched gene set are shown (Supplementary Table 5). (**c**) REST gene expression negatively correlates with tumor cell content and is therefore downregulated in siNETs. (**d**) Western Blot analysis of REST in normal tissue (tonsil and healthy small intestine) and siNET tissue of 3 samples from our cohort and 6 independent samples (*P* primary tumor, *MH* hepatic metastasis). (**e**) Marker genes that define EEC cell-type signature. (**f**, **g**) siNET gene expression was deconvoluted using NMF. (**f**) NMF factor 4 correlated with siNET marker genes, (**g**) resembles the EEC cell-type signature and (**h**) is associated with tumor cell content. (**i**) Expression of REST and Synaptophysin (SYP) in intestinal epithelial cells (**j**) Gene expression of EEC subtype markers in siNETs implicates enterochromaffin cells as potential cell-of-origin.

To further understand underlying molecular mechanisms of siNET tumor biology, we performed bulk RNA sequencing of our tumor samples (Fig. S1b). For deconvolution of tumor and non-tumor gene expression signatures, independent component analysis (ICA) was used. Independent component 3 (IC3) negatively correlates with tumor cell content (Fig. 3a). Gene set enrichment analysis of IC3 identified enrichment of target genes of *CDX2*, a transcription factor that is highly and exclusively expressed in gut epithelium^41,42^, emphasizing IC3 as a component that distinguishes tumor from adjacent normal tissue gene expression (Fig. 3b). Of note, CDX2 itself is expressed in tumors as well and does not correlate with tumor cell content reflecting its use as a marker of intestinal origin in case of cancer of unknown primary (Fig. S6). Interestingly, IC3 is depleted of *REST* (RE1-silencing transcription factor) target genes (Fig. 3b). Indeed, expression of the transcription repressor *REST* negatively correlates with tumor cell content and is thus depleted in tumors (Fig. 3c). Western blot analyses confirmed the absence of REST in siNETs (Fig. 3d).

*REST* is a master transcription repressor of neuronal genes and a proposed tumor suppressor in several malignancies like Wilms Tumor, triple negative breast cancer and small cell lung cancer^43–45^. Low expression of *REST* is a novel transcriptomic signature of siNETs. However, this signature is not explained by genomic alterations.

### Gene expression profiles of siNETs resemble enteroendocrine cells (EECs) and implicates them as the cell of origin

Beside a tumorigenic function, low expression of *REST* could also be inherited from the cell of origin of siNETs. Although it has been assumed since the early twentieth century that siNETs originate from EECs, the only evidence supporting this assumption is the morphological and immunohistochemical resemblance between the two^46,47^. While others have also hypothesized that siNETs originate from EECs using molecular data^48^ we have not encountered systemic cell of origin analysis that considered the cell types in the small intestine and EEC sub types. To elucidate the cell of origin, we performed non-negative matrix factorization (NMF) on the gene expression profiles of cell type-specific marker genes obtained from publicly available single-cell RNA sequencing (scRNA-seq) data of the human intestine^49^. Based on cell-type markers (Figs. 3e and S7a) we obtained four factors where factor 4 correlated with enteroendocrine marker genes and shows the highest similarity to the EEC gene expression signature (Figs. 3f,g and S7b). Additionally, this factor correlates with tumor cell content (Fig. 3h) thus implicating EECs as the cell of origin. Interestingly, not only the clinical biomarker of siNETs–Synaptophysin—followed the expected expression in EECs but also *REST* expression is low in EECs (Fig. 3i) suggesting that low expression of *REST* may be inherited from EECs, the cell of origin of siNETs.

Due to the low number of EECs from non-fetal, healthy tissue in the scRNA-seq data, a deconvolution using the EEC subtypes could not be performed, however the expression of typical EEC subtype markers^49^ (Fig. S7d) in the siNET samples showed high expression of *TPH1* (Fig. 3j)—a marker for enterochromaffin cells^50^. *TAC1* was also highly expressed in all but a single sample, further supporting the hypothesis that enterochromaffin cells are the EEC subtype of origin. Interestingly the sample that had low *TAC1* expression also had a high *NEUROG3* expression pointing at a progenitor signature, however, this is confounded by the fact that this patient was the only one in the cohort that received chemotherapy.

## Discussion

In this study we analyzed the genome and transcriptome of 39 well differentiated siNETs. We found that siNETs are in general mutationally quiet without clear driver mutations explaining the initial oncogenic transformation. *CDKN1B* mutations were previously reported as a driver albeit with a low frequency^4–8^. We detected only one *CDKN1B* mutation, however this could be due to a low sample size. By analyzing multiple samples from one patient Elias et al. demonstrated that even in one patient *CDKN1B* mutations occurred inconsistently between multiple primary tumors. These results together suggest that *CDKN1B* mutations are enriched in siNETs but cannot explain exclusively siNET formation. We did not observe any other frequent gene mutation.

In accordance with the literature, various chromosomal aberrations were detected in our siNET cohort, especially loss of chromosome 18. The functional relevance of chromosome 18 loss has been discussed longish and controversially with involvement of several tumor suppressors like *DCC*, Elongin A3, *SMAD2*, *SMAD4*, and *MIR1-2*, but so far, no conclusive results exist^12,51–53^. Elias et al. also demonstrated that 7 of 11 patients showed an inconsistency in chromosome 18 deletion across multiple primary samples and additionally, two more did not show chromosome 18 deletion^6^. In line with that finding, we also observed an inconsistency in chromosome 18 loss in paired samples in one of our patients (P1, primary tumor and metastasis). These results suggest that tumors benefit from chromosome 18 deletion, but it is not necessary for initial oncogenic transformation, as in the same patient, some primary tumors exhibit chromosome 18 loss, and some do not. Furthermore, we observed a reduced prevalence of chromosome 18 deletion in metastatic lesions compared to primaries, suggesting a non-essential role of chromosome 18 deletion in tumor progression.

In summary, siNETs display several chromosomal aberrations but do not possess any common driver mutations and can arise independently with no shared genetic background. This indicates that there is no early genetic event that clearly explains the initial oncogenic transformation from an EEC to a siNET cell for most cases. We assume that a larger region of healthy small intestine is subject to certain conditions that confer susceptibility to siNET generation. After this first step, observed genomic and chromosomic alterations provide selective advantage and allow progression and metastasizing after siNET formation. We looked for specific conditions that could predispose siNET development and found no evidence of bacterial infections, the genotoxin colibactin, telomere lengthening or loss of imprinting of *IGF2*. In three patients with multi-focal tumors, we found germline mutations in the FANC DNA repair pathway, strongly linked to HR DNA repair^34,35^, which could explain the high number of chromosomal alterations observed in siNETs. Consistent with our results, germline mutations in siNETs were previously described with a frequency of 9–11% and include mutations in *ATM*, *RAD51C*, *MUTYH*, and *BLM*^36,37^. Interestingly, all four genes are involved in DNA repair and partially interact with the FANC gene products^33,38–40^. Therefore, our results suggest a potential role of genetic predisposition in DNA repair-related pathways, although the precise way it operates remains to be elucidated.

We analyzed the SBS mutation signatures in both shared and non-shared mutations in primary and metastasis pairs from the same patient^22^. The defective HR DNA repair signature SBS3 only occurred in non-shared mutations, indicating that this mutational signature is a characteristic of late tumor stage. While we did not observe clear indications of large scale genomic instability in siNETs, elevated levels of the mutational signature of defective HR DNA repair in advanced siNETs is an interesting finding and a possible explanation why siNETs exhibit a high number of chromosomal alterations–especially in the metastatic state—and a more aggressive behavior over time with an increase of their Ki67 index^54^. Together, germline FANC mutations and somatic SBS3 signatures implicating defective DNA repair argue for considering novel therapeutic strategies, e.g. PARP inhibitors, and re-biopsies during the disease process to track the evolution of the tumor and adopt treatment decisions accordingly.

We further investigated possible tumor initiating events at the transcriptomic level. RNA sequencing revealed EECs as the cell of origin of siNETs. Although, this was assumed for a long time^46,47^, evidence besides histological analyses and immunostaining was lacking. By analyzing the transcriptome of intestinal EECs and our siNETs we detected a strong transcriptomic similarity arguing for EECs as the cell of origin. Further analysis provided evidence that serotonin producing enterochromaffin cells, an EEC subtype, are the cell of origin.

Additionally, we detected low expression of *REST* as a hallmark of siNETs. *REST*, a transcriptional repressor of neuronal genes, has been described as a tumor suppressor in several malignancies^43–45^. Also, for colon cancer a tumor suppressing function was described where a reduced *REST* expression resulted in enhanced activity of the PI3K/AKT/mTOR signaling pathway, the target of Everolimus, a main therapeutic agent in siNET treatment^55^. Beside the tumor suppressing function, *REST* is also a key player in regulation of transdifferentiation of epithelial malignancies: It was previously shown that downregulation of *REST* in hormone refractory prostate cancer and small cell lung cancer caused a transdifferentiation towards a neuroendocrine tumor state with expression of neuroendocrine markers like Synaptophysin^56,57^. Furthermore, our data suggests that low expression of *REST* could be inherited from the cell of origin as *REST* is already suppressed in EECs and observed genomic alterations did not explain low *REST* expression. Conducting scRNA-seq of both EECs and siNET cells would allow for more accurate analyses by identification of the transcriptomic differences between the two, however, this is complicated by the low number of EECs in the healthy epithelium. While this calls for strategies to enrich for the EEC population for targeted phenotypic characterization of the cell of origin, this is currently impeded by a lack of robust surface markers for EECs to enable enrichment. Furthermore, since most siNETs lack clear genomic drivers, we believe ongoing research should also explore epigenomic perturbations in siNETs compared to EECs—their cell of origin.

## Methods

### Clinical cohort

32 patients underwent surgery for siNETs or their metastases. The clinical characteristics are given in Supplementary Table 1. Pathological analysis and confirmation of G1/G2 NET diagnosis were performed following clinical routine. A tumor piece and adjacent normal tissue samples were collected at surgery and directly frozen in liquid nitrogen.

The collection of tissue samples for this study was approved by the Ethics Committee of the Charité–Universitätsmedizin Berlin (No EA1/229/17). All patients received in-person as well as written information about the study. Informed consent was obtained and documented by signing a document. All methods were performed in accordance with the relevant guidelines and regulations.

### Tissue homogenization

For preservation, the frozen tissues were transitioned into a minimum of 10 volumes of RNAlater™-ICE (Invitrogen™, ThermoFisher Scientific, MA, United States) at − 70 °C or − 80 °C. For the bead-based homogenization the tissue sections were transferred in Precellys Soft tissue homogenizing tubes (Soft tissue homogenizing CK14 Lysis kit; Bertin Technologies SAS, FRANCE) pre-filled with ceramic beads and lysis buffer. The samples were homogenized using the Precellys Evolution Homogenizer (Bertin Technologies SAS, FRANCE). The homogenizer was operated at 4500 rpm of 10 s run time and using the cooling-mode for RNA samples. For DNA samples three cycles of 20 s run time at 7500 rpm were used, and a 30 s break interval between the cycles.

### RNA extraction

For isolation of total RNA, the automated high-throughput magnetic-bead extraction MagMAX™ *mir*Vana™ Total RNA Isolation Kit (Thermofisher Scientific, Waltham, MA, United States) was used with the King Fisher Flex system. The Kit also contains reagents for efficient, complete digestion of DNA by TURBO™ DNase (2 U/μl) along with removal of the enzyme. RNA concentration was quantified by Qubit™ 3 Fluorometer (Invitrogen™, ThermoFisher Scientific, MA, United States) in combination with Qubit RNA BR Assay Kit (Invitrogen™, ThermoFisher Scientific, MA, United States) and the RNA quality was verified based on RNA Integrity Number (RIN) scores estimated using RNA Screen Tape System on the 2200 Tape Station (Agilent Technologies, CA, United States). For the library preparation using the Poly(A) mRNA enrichment a RIN score of at least 7 (RIN >  = 7) was required.

### Extraction of genomic DNA

Isolation of genomic DNA from tissues was done using the MagMAX DNA Multi-Sample Ultra 2.0 on the Thermo Scientific™ KingFisher™ Flex Duo system (Applied Biosystems, ThermoFisher Scientific, Waltham, Massachusetts, United States). Extracted genomic DNA was quantified with the Quant-iT double-stranded genomic Broad Range DNA assay (Invitrogen™, ThermoFisher Scientific, Waltham, Massachusetts, United States) on the FLUOstar^®^ Omega fluorescence plate reader (BMG Labtech, Ortenberg, Germany). DNA integrity and size in base pairs (bp) were analyzed on the Agilent™ 4200 TapeStation™ System with a Genomic DNA ScreenTape™ device (Agilent Technologies, CA, United States).

### WGS library preparation and sequencing

For library preparation the TruSeq^®^ Nano DNA Library Prep kit, including IDT for Illumina TruSeq DNA UD Indexes, (8 nucleotides; 96 Indexes; 96 Samples) (Illumina, CA, United States) was used. Library Preparation was performed according to the manufacturer’s instructions. Because of a genomic DNA input of 100 ng for the library preparation, the initial concentration was set between 15 and 25 ng/ul and initially sheared by sonication on the Covaris ME220 (Covaris Inc., Perkin Elmer, MA, United States) using the settings specified for a fragment size of 350 bp.

The preparation protocol was adapted to the Biomek i7 workstation (Beckman Coulter, CA, United States), including the PCR steps. Enrichment was performed using 7 cycles of PCR, according to the manufacturer’s recommendations. To assess the quality of the libraries, the fragment size was verified by checking the library size distribution (target insert size 550 bp) on an Agilent Technology Tape Station using the D5000 assay for analyzing double-stranded DNA molecules from 100 to 5,000 base pairs. The libraries were firstly quantified using Qubit 3.0 Fluorometer and additionally analyzed by the KAPA Library Quantification Kit (Roche, IN, United States), including NGS platform-specific library quantification primer on a Roche^®^ LightCycler 480.

Libraries were sequenced on the Illumina^®^ NovaSeq 6000™ system using the S4 reagent Kit (paired end; 2 × 150 bp reads). The number of libraries per flow cell was based on the desired minimum coverage for the individual sample type: 30 × for controls; 60 × for tumor samples.

### WTS library preparation and sequencing

For PolyA mRNA enrichment by the NEBNext Poly(A) mRNA Magnetic Isolation Module (New England Biolabs) an amount of 150 ng of high-quality RNA samples with a RIN score of at least 7 (RIN >  = 7) are required. NEBNext Ultra II Directional RNA Lib-Prep Kit for Illumina and the NEBNext Multiplex Oligos for Illumina Set 1 (96 Unique Dual Index Primer Pairs) (New England Biolabs) were used for the library preparation. All procedures were performed according to the protocols suggested by the manufacturers and adapted to the Biomek i7 workstation (Beckman Coulter, CA, United States). The fragmentation time and clean up conditions were set resulting in an insert size about 300 base pairs for the libraries. The concentration of the libraries was measured using Invitrogen Quant-iT dsDNA BR Assay (FisherScientific) and the microplate reader FLUOstar Omega (BMG Labtech, Germany) and analyzed on 4200 Tape Station (Agilent Technologies) using the High Sensitivity D1000 assay. Additionally, the libraries were analyzed by the KAPA Library Quantification Kit (Roche, IN, United States), including NGS platform-specific library quantification primer on a Roche^®^ LightCycler 480.

Libraries were sequenced on the Illumina^®^ NovaSeq 6000™ system using the S4 reagent Kit (paired end; 2 × 150 bp reads). The number of libraries per S4 flow cell was based on at least 100 million reads per transcriptome library.

### Western blot

NET tissue was intensively washed with PBS (Gibco), lysed with RIPA buffer (Thermo Fischer Scientific) containing a protease inhibitor cocktail (cOmplete™ Mini, Roche) and sonication (10 s, 60% intensity). The proteins were separated by SDS-PAGE (10–12% Tris-Glycin, WedgeWell™, Invitrogen) and transferred onto nitrocellulose membranes (0.2 µm, #1620112, Bio-Rad) that were blocked with 5% nonfat dry milk. Afterwards the membranes were incubated with primary antibodies against REST (1:2000, AK #22242, Proteintech) and α-tubulin (1:1000, #T9026, Sigma) overnight at 4 °C, followed by incubation with secondary anti-rabbit IgG antibody (1:10000, #111-035-045, Jackson Immuno Research) and secondary anti-mouse IgG antibody (1:10000, #AB_2340061, Jackson Immuno Research), respectively, for 1 h at room temperature. Detection was performed with SuperSignal™ West Dura Extended Duration Substrate (Thermo Fisher Scientific) using the Molecular Imager^®^ VersaDoc™ and quantified with Image Lab™ software (Bio-Rad). The presented western blot contains 3 samples from our cohort (P10, P11, and P13) and 6 independent samples (3 primary siNETs and 3 hepatic metastases of siNETs).

### Whole-genome sequencing alignment

Whole genome sequencing reads were aligned with the DKFZ alignment and QC workflow (v1.2.73-201, https://github.com/DKFZ-ODCF/AlignmentAndQCWorkflows). Briefly, the reads were mapped using *bwa mem* (v0.7.15)^58^ to the human reference genome build 37 (hs37d5) with a base quality threshold of 0. Next, the alignments were converted to BAM format and sorted by coordinates using *samtools* (v0.1.19)^59^ before marking duplicates using *sambamba* (v0.6.5)^60^ with compression set to 0.

### Small variant identification

#### Single nucleotide variant calling

Single nucleotide variants (SNVs) were called with the DKFZ SNV calling workflow (v1.2.166-3, https://github.com/DKFZ-ODCF/SNVCallingWorkflow). Briefly, *samtools* (v0.1.19)^59^ and *bcftools* (*htslib* v0.2.5^61^) were used to identify SNVs and germline mutations were marked by comparing tumor to matched control samples. The quality of each SNV was characterized by checking for overlap with repeats, DAC blacklisted regions, DUKE excluded regions, self-chain regions, segmental duplicate records from ENCODE^62^, and the presence of PCR or strand biases.

#### Insertion and deletion calling

Indels were identified using the DKFZ indel calling workflow (v2.4.1-1, https://github.com/DKFZ-ODCF/IndelCallingWorkflow). *Platypus*^63^ was used to identify indels and the internal confidence call was used for filtering indels with a score above 8.

#### Rescue of tumor-in-normal mutations

Tumor in Normal Detection Analysis (TiNDA, https://github.com/NagaComBio/TiNDA) was used as previously described in Ishaque et al.^30^ to rescue mutations that were filtered as germline due to contamination of adjacent normal tissue with tumor DNA. All TiNDA rescued clusters were manually checked, and in case that clustering did not correctly identify the tumor-in-normal cluster mutations were manually rescued.

#### Tumor mutational burden and hypermutated regions

The tumor mutational burden (TMB) was calculated as the number of somatic SNVs and InDels in coding regions divided over the summed lengths of distinct non-overlapping coding regions (35,345,952 bp) in Gencode v19 gene models.

Hypermutated regions which are indicative for kataegis events were identified by calculating the intermutational distance of SNVs. Genes that overlapped a region containing at least 6 SNVs with an average intermutational distance below 1000 bp were classified as hypermutated.

#### Structural variation calling

To identify structural variants the DKFZ Sophia workflow (v2.2.3, https://github.com/DKFZ-ODCF/SophiaWorkflow) was employed. Structural variation (SV) candidates were generated by *SOPHIA* (v35) on the basis of *bwa-mem* supplementary alignments. The candidates are further filtered by comparison to a background database of normal controls generated from over 3,000 patients. The GENCODE V19 gene annotations were used to annotate SVs within genic regions and SVs were filtered for a minimum ‘clonalityRatio’ of 0.1 for both breakpoints.

#### Copy number aberrations

Copy number aberrations (CNAs) were called using the DKFZ ACEseq Workflow (v1.2.8-4, https://github.com/DKFZ-ODCF/ACEseqWorkflow). *ACEseq*^64^ determines tumor ploidy, chromosomal copy numbers, and tumor cell content (tcc) based on coverage ratios and B-allele-frequencies (BAF) of heterozygous SNPs. Genome segmentation was estimated by integration of previously identified SVs.

All purity and ploidy results were manually checked and corrected if the estimated tcc and ploidy did not sufficiently fit the BAF and coverage profiles.

#### Frequency of copy number aberrations

To calculate the frequency of CNAs across multiple samples the *ACEseq* output was transformed to an appropriate format to be processed using *GISTIC2.0* (v2.0.23)^65^ with the provided hg19 reference genome file.

#### Mutational signature analysis

Mutational signature analysis for SNVs and indels was performed using *YAPSA* (v1.19.0)^66^. The mutational catalogue was build using the *BSgenome.Hsapiens.UCSC.hg19* (https://doi.org/10.18129/B9.bioc.BSgenome.Hsapiens.UCSC.hg19) (v1.4.3) as reference and decomposed jointly for all samples in ‘consensus’ mode. *YAPSA* is based on the COSMIC Mutational Signatures v3.0 and only the real signatures were included (i.e. artificial signatures are not taken into account) in the decomposition together with their optimized signature cutoffs provided by the package.

To evaluate the presence of the Colibactin signature it was downloaded from the original publication^67^ and added to the signature catalogue used by *YAPSA*, and the previously described analysis was repeated with the same parameters.

To analyze the copy number signatures from Steele et al.^23^, copy number profiles were computed using *ASCAT* (v3.0.0)^68^ to match the copy number calls used to extract these signatures. Briefly, *ASCAT* was run for WGS by extracting allele counts at specific loci with *ascat.prepareHTS* and calculating logR and BAF values. logR values were corrected with *ascat.correctLogR*, and segmentation performed with *ascat.aspcf*. Copy numbers and purities were calculated with *ascat.runAscat* limiting the *max_ploidy* to 2.5 and setting *gamma* to 1 as per the documentation. The relevant reference files for loci, alleles, GC correction and replication timing were downloaded for hg19 following the documentation.

Copy number signatures were analyzed using the *SigProfiler* suite. The mutational catalogue was extracted using *CNVMatrixGenerator.generateCNVMatrix* from *SigProfilerMatrixGenerator* (v1.2.12)^69^ and the 21 non-artefactual CN signatures were assigned using *Analyzer.cosmic_fit* from *SigProfilerAssignment* (v0.0.13)^70^.

#### Telomere content analysis

To analyze telomere content of tumor and normal tissue, TelomereHunter (v1.1.0)^71^ was run with both, tumor and control bam files. To correct for the tumor cell content (tcc) of tumor samples the estimated telomere content was adjusted using the formula:

$$telomere_{corrected} = \frac{{telomere_{tumor} - telomere_{control} *\left( {1 - tcc} \right)}}{tcc}.$$

#### Tumor heterogeneity analysis

To analyze tumor heterogeneity using multiple samples of the same patient, small variant (SNVs and indels) positions were evaluated for clonality. First, genomic positions of small variants across all tumor samples of a patient were merged, and pileups generated using *samtools mpileup* (v1.13)^72^. The variant allele fraction was calculated for each sample and corrected for tumor copy number and tumor cell content (tcc) to calculate the predicted number of mutated alleles using the formula:

$$Alleles_{mutated} = VAF*\frac{CN*tcc}{{CN*tcc + 2*\left( {1 - tcc} \right)}}*CN.$$

where $Alleles_{mutated }$ refers to the predicted number of mutated alleles, previously referred to as the mutation copy number^30^. All SNVs with coverage between 20 and 250 reads (to exclude lowly and highly covered regions) were split into metastasis-specific, tumor-specific, and shared mutations per patient depending on athreshold of 0.5 Alleles_mutated_. The resulting clusters were filtered for a minimum size of 100 SNVs (to avoid noisy deconvolution) and mutational signature analysis was performed as previously described. Difference in normalized signature exposure between the clusters was tested using a Kruskall-Wallis rank sum test. To further validate the results using a different mutational signature deconvolution method we also applied *SigProfiler Assignment* (v0.1.8)^70^ using the *Analyzer.cosmic_fit* function.

#### Germline predisposing mutations

To identify potential tumor predisposition from germline mutations or single nucleotide polymorphisms (SNPs) identified in normal whole genome sequencing samples we used *CPSR* (*PCGR* v1.0.3)^73^ to identify pathogenic and likely pathogenic variants using the CPSR exploratory cancer predisposition panel (panel-ID 0) consisting of 433 genes curated from the TCGA’s Germline Study, COSMIC’s Cancer Gene Census v100, Genes from all Genomics England PanelApp panels for inherited cancers and tumor syndromes, as well as DNA repair genes, and additional genes deemed relevant for cancer predisposition (i.e. contributed by CPSR users).

#### Transposable element insertion

*xTea* (v0.1.7)^74^ was used to evaluate tumor samples for Transposable element (TE) insertions. The tumor samples were processed in case–control mode of *xtea_hg19* analyzing L1, Alu, and SVA insertions. The reference genome was the same as for WGS alignment and as gene annotation the GENCODE release 33 for GRCh37 was used.

#### Foreign DNA detection

To detect foreign DNA, such as bacterial or viral sequences, we used *Kraken2* (v2.1.2)^75^ and *Bracken* (v2.6.1)^76^. Briefly, the default *kraken2* database was built using *kraken2-build* with the *standard* flag. All read pairs with at least one unaligned read were extracted from the BAM-files using *samtools* (v1.14)^59^ and converted to fastq files for read 1 and 2 using *bedtools* (v2.30.0)^77^. *Kraken2* was run in paired-end mode and the output and report with aggregated counts per clade written to file. Genus abundances were estimated with *bracken* after building a database with *k*-mer length of 35 and read-length of 150 (matching our sequencing protocol) with *bracken-build*.

#### RNA sequence alignment and gene expression quantification

The DKFZ RNAseq workflow (v1.3.0-1, https://github.com/DKFZ-ODCF/RNAseqWorkflow) was used to align the reads from RNA sequencing and quantify gene expression. Briefly, reads were aligned against a STAR index generated for reference genome hs37d5 and aligned in 2 pass mode using *STAR* (v2.5.3a)^78^. The alignment was converted to BAM format and sorted by coordinates using *samtools* (v1.6)^59^ and duplicate reads were marked with *sambamba* (v0.6.5)^60^ but not removed. To quantify gene expression *featureCounts* (*Subread* v1.5.1)^79^ was run with the GENCODE V19 gene model, counting reads non-strand specific over exon features, without exclusion of duplicate reads and only considering pairs with two uniquely mapping fragments (quality score of 255). For transcripts per million (TPM) calculation, gene counts on the X, Y, and MT chromosomes as well as rRNA and tRNA were excluded from library size estimation to avoid biases.

#### Unsupervised gene expression analysis

To analyze the tumor gene expression in an unsupervised manner, independent component analysis (ICA) was employed. First, gene expression counts were filtered for protein-coding genes that were detected in at least 3 samples. The *rlog* transform from *DESeq2* (v1.34.0)^80^ was used with default parameters to stabilize the variance. The 1000 most variable genes were selected and the *FastICA* implementation from *scikit-learn* (v1.0.1)^81,82^ was used to generate a latent space with 7 components.

#### Gene set enrichment analysis

To analyze the different gene signatures identified via ICA gene set enrichment analysis (GSEA)^83^ was run using ICA gene weights of each factor as ranking criterion. *MIGSA* (v1.18.0, https://bioconductor.org/packages/3.14/bioc/html/MIGSA.html) was used to download the gene set collections *ChEA_2016, ENCODE_and_ChEA_Consensus_TFs_from_ChIP-X, TRRUST_Transcription_Factors_2019, ClinVar_2019, MSigDB_Hallmark_2020,* and *MSigDB_Oncogenic_Signatures* from Enrichr^84^ (http://amp.pharm.mssm.edu/Enrichr). *gseGO* and *gseKEGG* from *clusterProfiler* (v4.2.0)^85^ and *gsePathway* from *ReactomePA* (v1.38.0)^86^ were used to analyze biological process gene ontologies, Kegg pathways, and Reactome pathways, respectively. All prior downloaded gene sets were analyzed using *GSEA* from *clusterProfiler*. The Benjamini–Hochberg procedure was used for multiple testing correction per gene set collection as implemented in *clusterProfiler*.

#### Allele specific expression analysis of IGF2

To evaluate the loss of imprinting (LOI) at the IGF2 locus, we estimated the allele-specific expression (ASE) using heterozygous SNPs identified from whole genome sequencing data. Heterozygous SNPs were selected from the *ACEseq* output (see section Copy number aberrations) and filtered to be within twofold of the average coverage of the respective sample and within a protein coding exon (GENCODE V19) using *bcftools* (*htslib*^61^ v1.16). SNPs were sorted, normalized (without removing duplicates), and converted to compressed BCF before concatenating all chromosomes per sample to a compressed VCF using *bcftools*. The Genome Analysis Toolkit (v4.3.0.0)^87^ *ASEReadCounter* module was used with a minimum mapping quality of 255 and a minimum base quality of 10 to estimate the ASE on the previously identified SNPs. The results were filtered by removing SNPs with a depth below 10, more than 3 ‘otherBases’, more ‘otherBases’ than ‘altCount’ and ‘refCount’, and all HLA genes. SNPs were then aggregated per gene by averaging the expression ratio of the allele with the most counts i.e. $\frac{{counts_{allele} }}{{counts_{total} }}$.

#### Cell of origin identification

To identify the cell of origin, public scRNA-seq data was used to compare tumor gene expression to small intestinal cell-type signatures. Normalized gene expression for epithelial cell lineages was downloaded from www.gutcellatlas.org^49^. The data was subset to only include healthy, non-fetal samples and rare cell types were aggregated to their parental lineage (i.e. all enteroendocrine cells were merged, Goblet cell subtypes were merged, Stem cells and transiently amplifying cells were merged). The genes were subset to protein-coding genes identified in the tumor gene expression analysis and the top 10 marker genes per cell type were identified by using the first ranking genes using the *rank_genes_group* function with ‘wilcoxon’ as method and then filtering for a minimum log-fold change of 2 using *filter_rank_genes_groups* from *scanpy* (v1.9.1)^88^.

The *rlog*-transformed tumor gene expression was subset to the previously selected marker genes, normalized using the *normalize* function and decomposed into 4 factors using *NMF* (both *scikit-learn* v1.0.1) using an L1-ratio of 0 and *alpha_W* of 10^−3^. The cosine similarity was calculated for the NMF factors and the cell type-averaged single-cell gene expression signatures.

## Supplementary Information

Supplementary Figures.

Supplementary Table 1.

Supplementary Table 2.

Supplementary Table 3.

Supplementary Table 4.

Supplementary Table 5.

## Data Availability

Single-cell gene expression data of small intestines was obtained from www.gutcellatlas.org (49). Aggregated non-sensitive results are available as tab-separated value tables to Zenodo (10.5281/zenodo.15084372).

## Code availability

All code used to process aggregated results can be downloaded as Jupyter Notebooks from GitHub: https://github.com/HiDiHlabs/siNET_omics_analysis.
