# Supplementary material for: Small intestinal neuroendocrine tumors lack early genomic drivers, acquire DNA repair defects and harbor hallmarks of low REST expression
Source: Sci Rep. 2025 May 23;15:17969. doi: 10.1038/s41598-025-01912-4 (PMC12102166; doi:10.1038/s41598-025-01912-4)
Supplement: Supplementary file 1 — Supplementary Figures. [file 41598_2025_1912_MOESM1_ESM.docx]

# Supplementary Tables

See Excels

# Supplementary Figures


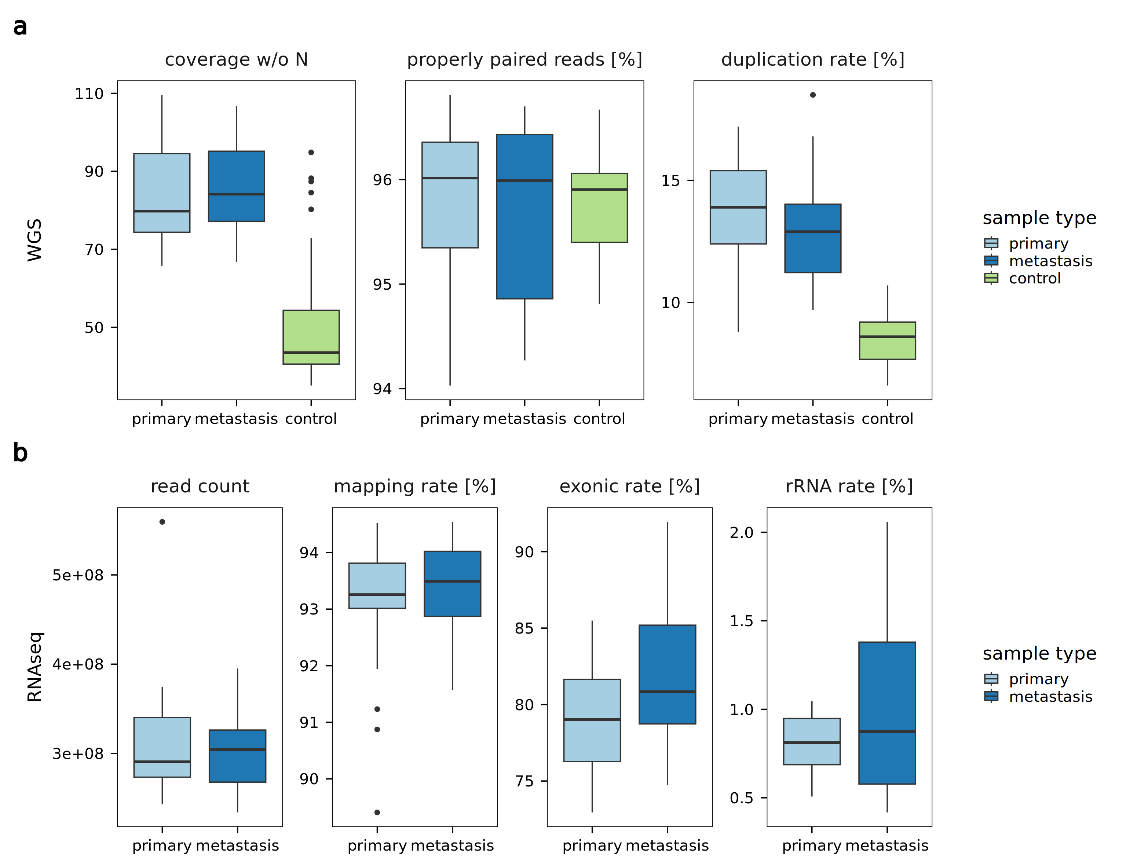


Figure S 1: **Quality control metrics** for **a)** whole genome and **b)** RNA sequencing.


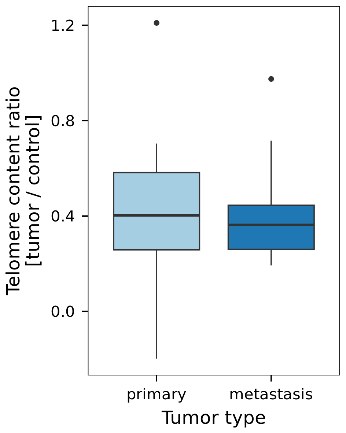


Figure S 2: **Telomere content of siNETs.** The tumor cell content-corrected telomere content in relation to the telomere content of control tissue in primary and metastases is shown.


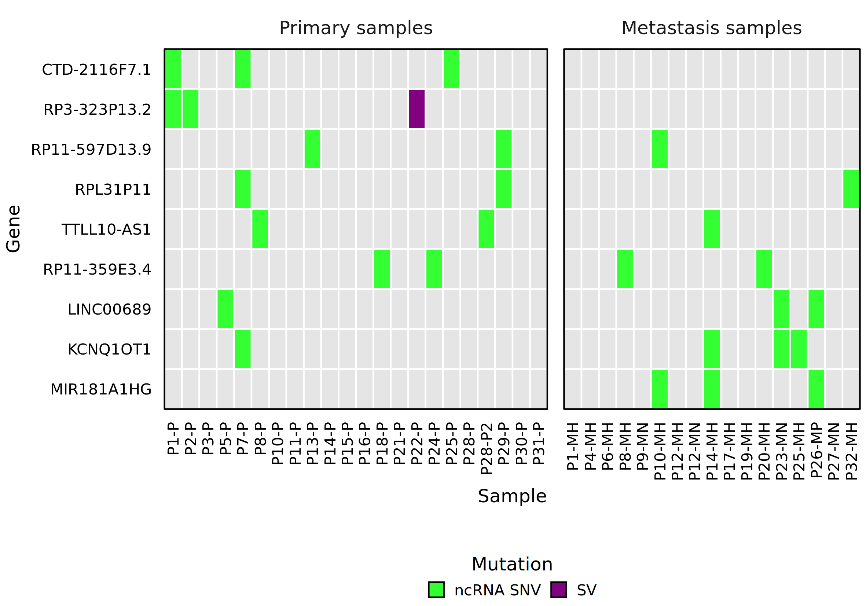


Figure S 3: **Recurrent mutations of noncoding genes.** Recurrent mutations (3 or more patients) of noncoding genes across all samples.


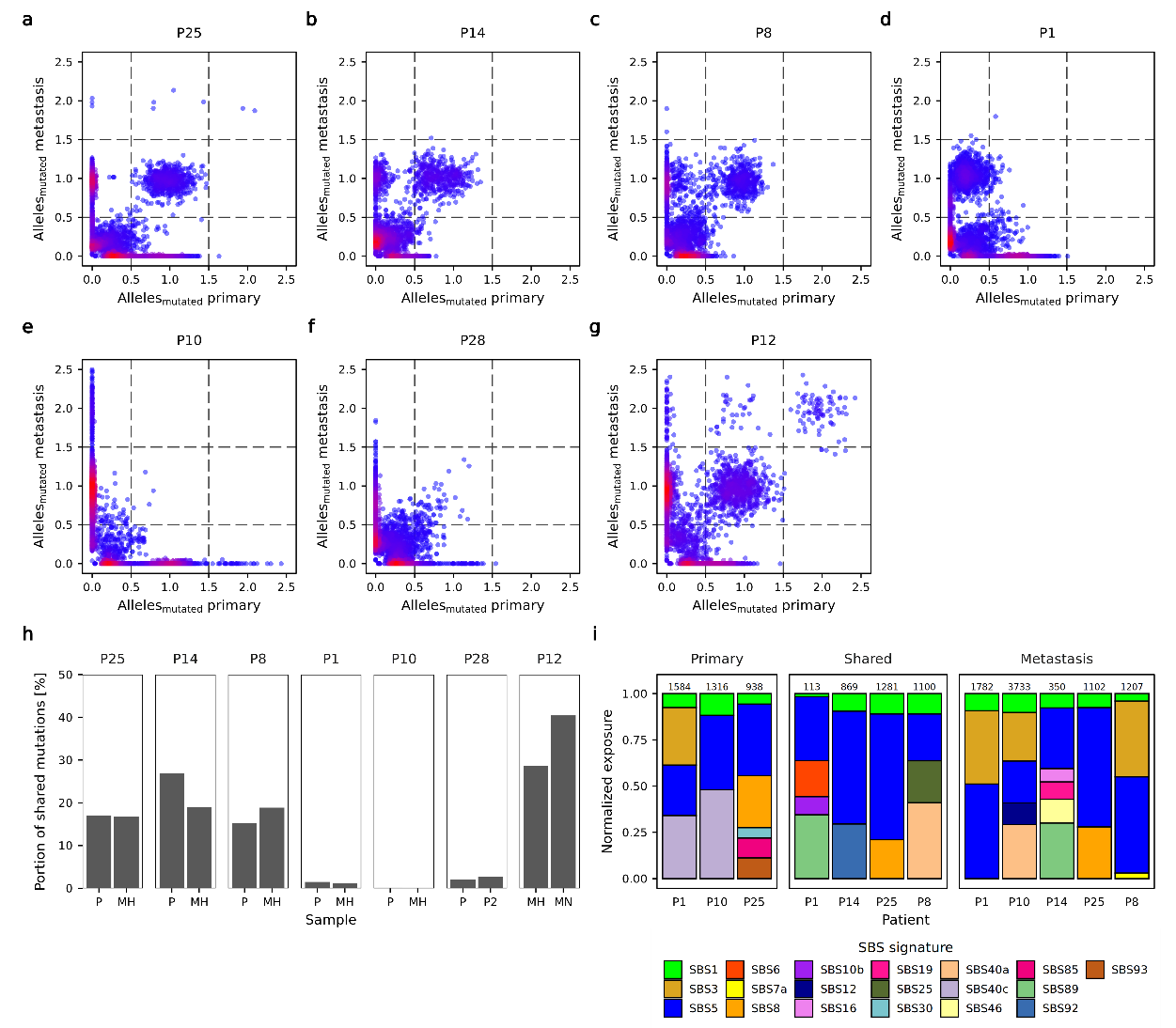


Figure S 4: **Shared mutations of paired samples. a-g)**Shared and sample specific mutations are shown for all tumor pairs of this cohort (compare Fig.2). **h)** Quantification of the shared mutations as ratio of all mutations per sample. **i)** Mutational signatures of primary-specific, metastasis-specific and shared SNVs obtained by SigProfiler Assignment.


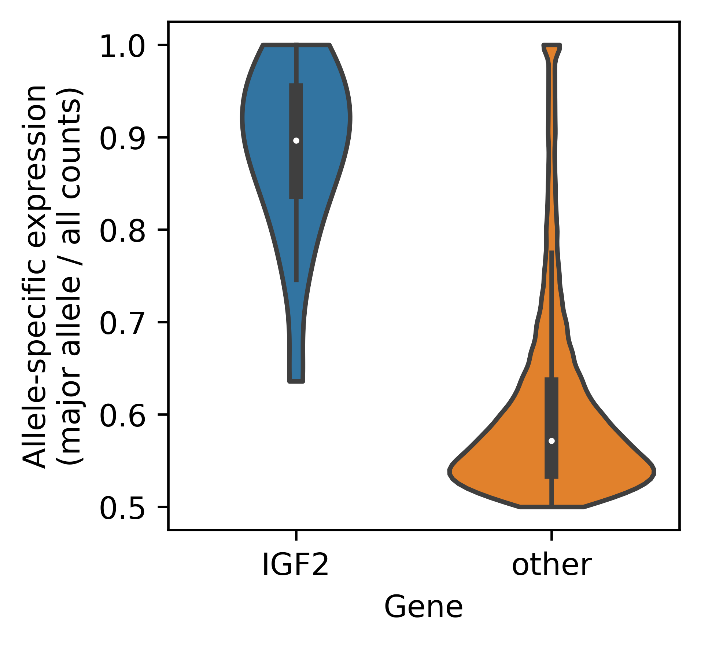


Figure S 5: **Allele-specific expression of IGF2.** The allele-specific expression across all samples for IGF2 and all other genes. As most genes are not under imprinting control, their level of allele-specific expression would be indicative of loss of imprinting (LOI). Most samples exhibit allele specific expression of IGF2 suggesting that imprinting is intact.


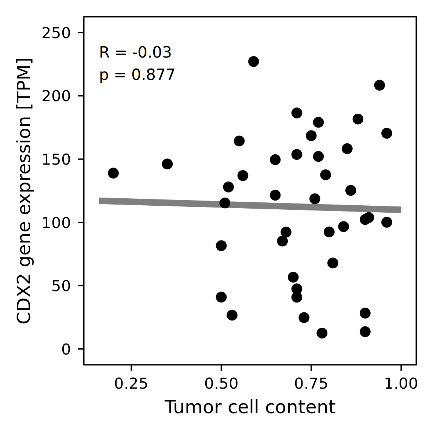


Figure S 6: **Gene expression of CDX2.** Gene expression of CDX2 in transcripts per million (TPM) in correlation to tumor cell content.


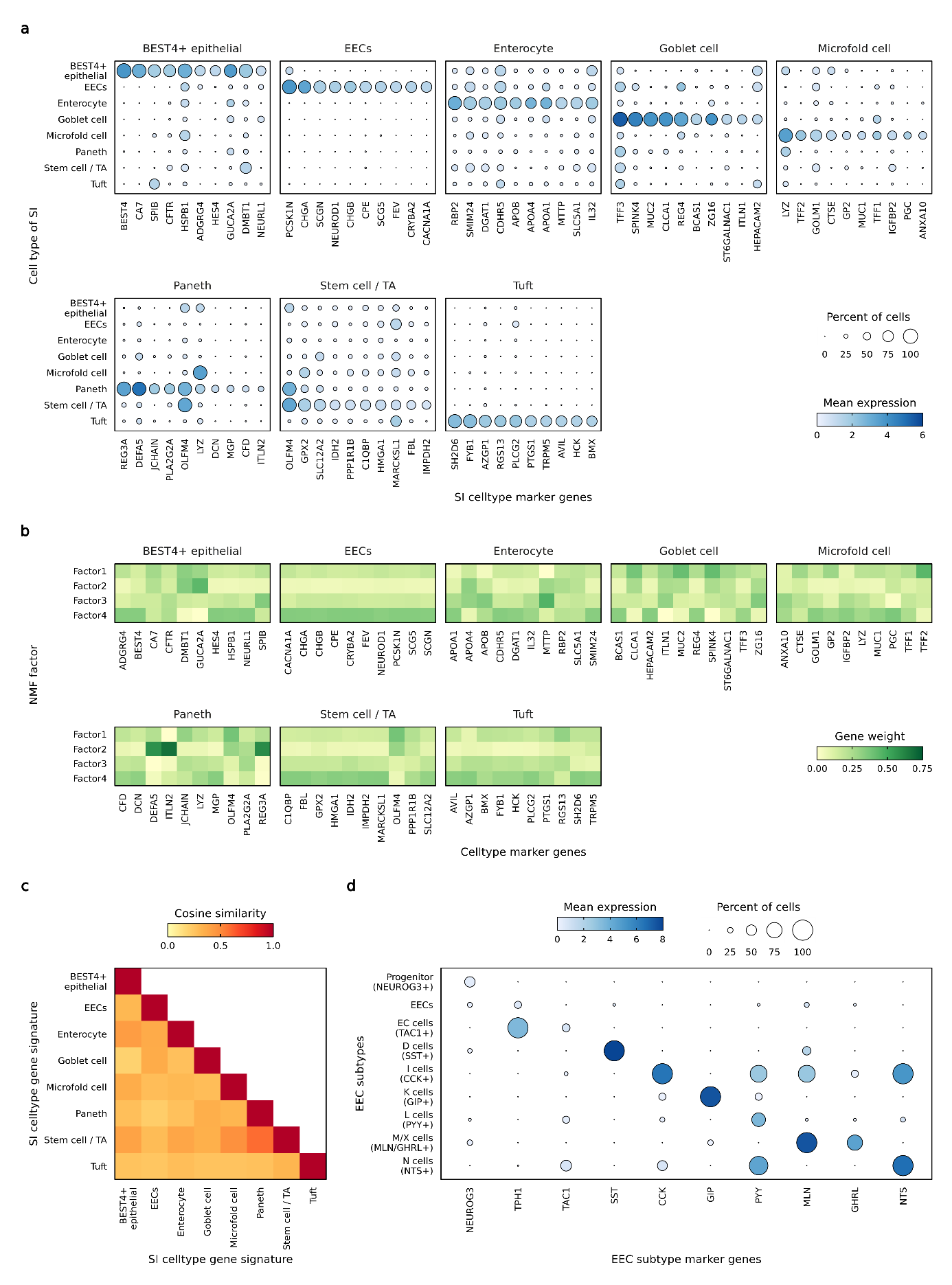


Figure S 7: **Cell-type markers and signatures of small intestinal cell types.** **a)** Gene expression of the marker genes for each small intestinal cell type. **b)** Gene weights of the NMF factors per cell-type marker gene show enrichment of EEC markers in factor 4. **c)** Similarity of small intestinal cell-type signatures. **d)** Marker gene expression of EEC subtypes.
